# Performance of bat-derived macrophages at different temperatures

**DOI:** 10.3389/fvets.2022.978756

**Published:** 2022-09-09

**Authors:** Monika Nemcova, Veronika Seidlova, Jan Zukal, Heliana Dundarova, Katerina Zukalova, Jiri Pikula

**Affiliations:** ^1^Department of Ecology and Diseases of Zoo Animals, Game, Fish and Bees, University of Veterinary Sciences Brno, Brno, Czechia; ^2^Institute of Vertebrate Biology, Czech Academy of Sciences, Brno, Czechia; ^3^Department of Botany and Zoology, Masaryk University, Brno, Czechia; ^4^Institute of Biodiversity and Ecosystem Research, Bulgarian Academy of Sciences, Sofia, Bulgaria; ^5^CEITEC-Central European Institute of Technology, University of Veterinary Sciences Brno, Brno, Czechia

**Keywords:** Chiroptera (bats), *in vitro* model, hibernation, phagocytic activity, temperature-dependent proliferation, daily torpor, macrophage biology

## Abstract

Heterothermy, as a temperature-dependent physiological continuum, may affect host-pathogen interactions through modulation of immune responses. Here, we evaluated proliferation and functional performance of a macrophage cell line established from the greater mouse-eared (*Myotis myotis*) bat at 8, 17.5, and 37°C to simulate body temperatures during hibernation, daily torpor and euthermia. Macrophages were also frozen to −20°C and then examined for their ability to proliferate in the immediate post-thaw period. We show that bat macrophages can proliferate at lower temperatures, though their growth rate is significantly slower than at 37°C. The cells differed in their shape, size and ability to attach to the plate surface at both lower temperatures, being spheroidal and free in suspension at 8°C and epithelial-like, spindle-shaped and/or spheroidal at 17.5°C. While phagocytosis at temperatures of 8 and 17.5°C amounted to 85.8 and 83.1% of the activity observed at 37°C, respectively, full phagocytic activity was restored within minutes of translocation into a higher temperature. Bat-derived macrophages were also able to withstand temperatures of −20°C in a cryoprotectant-free cultivation medium and, in the immediate post-thaw period, became viable and were able to proliferate. Our *in vitro* data enhance understanding of macrophage biology.

## Introduction

Heterothermic bats have adapted to high variation in all bodily functions (1–3), allowing them to save energy and water when ambient temperatures are low, or food is scarce (4–6). One such adaptation, their capacity to hibernate or utilize daily torpor, is characterized by complex behavioral and physiological changes that enable them to survive seasonal climatic extremes (2, 7–9). Torpor bout length and frequency of arousals must balance the physiological costs and benefits of euthermic periods (10, 11). If the torpor bout is too long, pathogenic agents may infect the organism unchecked (12). On the other hand, bats may deplete energy reserves during too frequent arousals, leading to emaciation and death (13, 14).

Both innate and adaptive immune functions and defense mechanisms against pathogenic agents are modulated by body temperature in heterothermic bats (7, 15–17). For example, while the body temperature of vespertilionid and rhinolophid bats varies from 10 to 25°C during daily torpor and 0–12°C during hibernation, normothermic temperatures of active bats are much higher than 30°C (18, 19). Thus, temperate region insectivorous bat species that decrease their body temperature close to the ambient temperature of a hibernaculum may not be fully competent to control infections during torpor bouts (7, 20). Further, there may also be a significant drop in white blood cell/phagocytic cell count during hibernation, driven by a clearance of leukocytes from circulation, meaning that the functions borne by these cells (monocytes, macrophages, and other phagocytes) may not be available at sites of infection and/or damage. These cells will only be fully restored during arousals and/or during the non-hibernation season (7, 21). Periodic arousals of hibernating animals may thus play an important role in strengthening immune responses, enabling bats to defeat pathogens that may have infected them prior to or during the inactive period (22).

As professional phagocytes, macrophages are highly efficient in the cellular process known as phagocytosis (23). Macrophages are long-lived tissue-resident cells that recruit from blood monocytes and reside in all tissues of the vertebrate body (24). These cells constitute the mononuclear phagocyte system, an evolutionarily ancient part of the immune system, and are critical drivers of both innate and adaptive immunity (25, 26). Macrophages are known to have dual pathways of activation, either promoting or inhibiting inflammation, defense against pathogens and tissue homeostasis, repair, and healing (24, 27). Interestingly, regarding the balance of these dual activation pathways, a high level of anti-inflammatory responses has been observed in bat macrophages (28).

While monocytes are spheroidal, macrophages differentiated in tissues take a variety of shapes, depending on the tissue of residence and its microenvironmental mechanical forces, biochemical milieu, oxygen supply, pH, and expression of genes (29, 30). Tissue macrophages typically show an ability to adhere to glass and/or plastic surfaces *in vitro* and are quick to change their function in response to tissue microenvironmental cues (26, 27).

Bats represent a unique model for studying temperature-dependent physiological changes and defense responses at the community, population, organismal, organ, cellular and/or molecular levels during repeated cycles of torpor and arousal (17, 31–36). As bats are wildlife reservoirs for many emerging pathogens, there is a need for studies into multiple aspects of bat-borne infections (37–40), including the impact of hibernation on the pathogenesis of diseases, which is still not fully understood (37). However, bats are strictly protected in Europe (Agreement on the Conservation of Populations of European Bats, EUROBATS), meaning that *in vivo* experiments at the organismal level are not possible. In such cases, the availability of bat-derived cell culture models would be highly advantageous as they could replace the experimental animals with specific immune cells. Moreover, the use of cell culture models would allow for the use of experimental designs, sample collection methods and measurement conditions that may not be feasible when using experimental animals (41). As an example, blood samples cannot be collected from bats in torpor and a re-warming period of around 30–60 min would be required before it would be possible (42). However, in this case, the parameters measured would not be representative of values expected when the bat is in a lower-temperature physiological state.

Here, we evaluate the performance of macrophages derived from the greater mouse-eared bat (*Myotis myotis*) in culture conditions simulating the body temperatures of bats during hibernation, daily torpor and euthermia. Bat-derived macrophages were also frozen to −20°C and examined in terms of their viability and ability to proliferate in the immediate post-thaw period. We predict that (1) the controlled conditions of cell cultures can be used to simulate body-temperature-dependant changes at morphological and functional cellular levels, and (2) different temperature-induced changes will be reversible in macrophages.

## Materials and methods

### Bat-derived cell culture

The greater mouse-eared bat macrophage cell line was used in the present study. The provenance of the cell line is described in He et al. (43). Cells were identified by mRNA expressing transmembrane 9 superfamily protein using RT-PCR reaction with primers F: GCTCTCTTAACCTGTCCTCCG and R: CTCTGTTCAGCCGGTTGTTG. To obtain enough cells of the same passage for individual experiments, the macrophages were cultured in T_75_ flask tissue culture (TPP Techno Plastic Products AG, Schaffhausen, Switzerland) at 37°C in a humidified atmosphere containing 5% CO_2_ prior to the experiments. The experimental macrophage stock was then prepared using DMEM-F12 1:1 medium, supplemented with 10% fetal calf serum (FCS), 100 IU/ml penicillin and 100 μg/ml streptomycin (ATB; Biosera, Boussens, France).

### Cell counting; experimental runs and repetitions

During the experiments, cells were enumerated using a Fast-Read 102^®^ plastic counting chamber (Kisker Biotech GmbH & Co.KG, Steinfurt, Germany). Non-adherent cells and cells detached from the cultivation surface using a 0.5% trypsin solution (Sigma-Aldrich, St. Louis, Missouri, USA) were counted individually. Only regular, round-shaped cells were considered as viable. All experiments were performed in 3 runs (replicates) with 3–5 repetitions.

### Experimental assays

Prior to each experiment, cells were detached from the cultivation surface using a 0.5% trypsin solution, after which they were washed twice with a medium containing DMEM-F12 1:1 with 10% FCS and centrifuged (Hermle Z326K, Gosheim, Germany) at 160 g for 6 min. The cell pellet was then re-suspended in new medium, either DMEM-F12 1:1 with 10% FCS and 1% ATB for counting and freezing or FluoroBrite™ DMEM (Gibco™ Thermo Fisher Scientific, Waltham, Massachusetts USA) with 10% FCS and 1% ATB for phagocytosis assays. Cells in suspension (i.e., 5,000 cells/ml of medium for counting, freezing and visualization of red zymosan or 100,000 cells/ml of medium for phagocytosis assay) were then placed onto test plates. Experimental designs to assess the performance of bat-derived macrophages in terms of growth rate and phagocytic functioning under specific culture conditions were as follows:

### Experiment 1: Cell growth at different temperatures

Each assay started with 10,000 suspended cells (2 ml) per well in six-well plates (TPP Techno Plastic Products AG, Schaffhausen, Switzerland). The cells were then cultivated at three different temperatures, i.e., 8, 17.5, and 37°C, under a 5% CO_2_ humidified atmosphere (myTemp Mini CO_2_ Digital Incubators, Benchmark Scientific, Sayreville, New Jersey, USA, for 8 and 17.5°C cultivation; CellCulture CO_2_ ESCO, Singapore, for 37°C cultivation) and counted daily for 6 days. Each day, two wells were reserved for staining and microscope cell photography. The plates were then centrifuged at 130 × g, the old medium removed, and the cells washed with phosphate buffered saline (PBS; Sigma-Aldrich, St. Louis, Missouri, USA), after which they were fixed using 4% formaldehyde diluted in PBS. Next, the cells were washed by gentle shaking for 5 min at room temperature, once with 0.1% triton in PBS and twice with PBS. Cells were then stained using Alexa Fluor™ 488 Phalloidin (Invitrogen™ Thermo Fisher Scientific, Waltham, Massachusetts, USA) and Antifade Mounting Medium with DAPI (Vector Laboratories, Burlingame, California, USA), according to the manufacturer's instructions, and visualized and photographed with the Cytation 1 microscope imaging multi-mode reader (BioTek, Winooski, Vermont, USA) using the DAPI 377/447, GFP 469/525 channels, and brightfield. As a negative control we used composite images obtained capturing untreated cells or cells treated with the Antifade Mounting Medium with DAPI in all fluorescent channels (in DAPI for calibration of Cytation 1 and in Texas red and GFP obtained under the same position as an evidence that there is no autofluorescence). Autofocusing images were also captured in the bright field every 2 min. Using the alignment of images we created a video sequence to observe movements of cells and changes of the cell culture at 37°C.

Cells were also captured and processed for size- and circularity in cell analysis under the bright field of a Cytation 1 microscope fitted with a size 4 Olympus objective, No. 1220519. The numbers of both adherent and in-suspension cells evaluated for each condition ranged from 57 to 111 cells. The morphometric analysis was performed using the Cytation 1 software. Circularity was calculated using a standard ellipse formula, where 1 = a perfect circle. Object area, calculated in μm^2^, was determined by counting the pixels inside the object contour and multiplying this count by a conversion factor calculated based on μm^2^ = (X-axis pixel length) × (Y-axis pixel length). Object size was then calculated as size in μm determined by fitting an ellipsis equation on the object contour.

### Experiment 2: Cell attachment and growth parameters

In the second assay, cells in six-well plates containing 10,000 cells per well were preincubated at 37°C for 24 h, which allowed the cells to attach to the plate surface. The cell count was then determined, and the plates relocated into incubators set at test temperatures of 8, 17.5, and 37°C. The cells were then enumerated every 24-h for the next 5 days.

### Experiment 3: Freezing and cell survival

In this experiment, macrophages were exposed to a freezing temperature of −20°C in a standard culture medium of DMEM-F12 with 10% FCS and ATB with no cryoprotectant added. Six-well plates with (A) 10,000 cells per well in suspension immediately after passaging or (B) 10,000 cells per well attached after 24 h pre-incubation at 37°C were kept in a freezer for 6 days, then allowed to thaw gradually at room temperature (21°C) before being enumerated. The frozen-thawed cells were also centrifuged, re-suspended in fresh medium, placed into new six-well plates, and cultured for 7 days under a humidified atmosphere of 5% CO_2_ at 37°C to examine their viability and re-growth after exposure to freezing temperatures.

### Experiment 4: Phagocytosis assay

To validate macrophage cell line functionality in terms of comparability with *ex vivo* phagocytosis, parts of the cell suspension, i.e., samples of 25,000 cells/well in 96-well plates (Corning Incorporated, Corning, New York, USA) replicated twice with 5 repetitions, were divided during cell passaging into experimental (temperature pre-incubated) plates. These cells were processed according to the luminescence-measuring protocol to evaluate phagocytosis through respiratory burst as described in Pikula et al. (17). Briefly, apart from the cell suspension the reaction mixture contained luminol (Sigma-Aldrich Merck KGaA, Darmstadt, Germany) dissolved in borate buffer, and Zymosan A (Sigma-Aldrich Merck KGaA, Darmstadt, Germany). Zymosan A concentration in the reaction mixture was 0.25 mg/ml. Chemiluminescence kinetics were measured for 2 h at 25°C using a Cytation 3M reader (BioTek Instruments, Inc., Winooski, VT, USA).

### Experiment 4a: Time dependent changes in phagocytosis (phagocytosis kinetics) under optimal phagocytic conditions after preincubation at test temperatures

Cells (10,000/well) were passaged into black 96-well plates in FluoroBrite DMEM supplemented with 10% FCS and ATB and incubated at 37°C overnight to allow attachment to the plate surface. The cells were then washed with PBS and the medium replaced with new FluoroBrite DMEM without FCS and ATB. Individual plates were then relocated into incubators set at test temperatures of 8, 17.5, and 37°C for 24 h. The cells were then counted and the medium replaced with Live Cell Imaging Solution (Gibco™ Thermo Fisher Scientific, Waltham, Massachusetts USA) supplemented with 15 mM dextrose (Merck, Sigma-Aldrich, Germany). The cells were then treated with pHrodo™ Green Zymosan BioParticles™ Conjugate for Phagocytosis (Invitrogen™ Thermo Fisher Scientific, Massachusetts, USA), prepared and diluted in Live Cell Imaging Solution as described by the manufacturer. At the same time, control cells were also prepared in zymosan-free Live Cell Imaging Solution supplemented with 15 mM dextrose. The Zymosan-treated and control cells were then prepared and time-dependent changes in phagocytosis evaluated by measuring fluorescence (Ex/Em) at 485/528 nm on a Cytation 1 Multi-Mode Reader every 7 min for 2 h at a temperature of 27°C. The pHrodo Zymosan was added to macrophages at their respective temperatures before time point 0 and the plates were transferred for measurement in Cytation 1 within a min.

### Experiment 4b: Extension of phagocytosis at different temperatures

Cells (10,000 cells/well) in six-well plates were incubated in DMEM supplemented with 10% FCS and 1% ATB for 48 h at test temperatures of 8, 17.5, and 37°C, after which a 10x solution of pHrodo™ Red Zymosan Bioparticle™ Conjugate for Phagocytosis (Invitrogen™ Thermo Fisher Scientific, Massachusetts, USA) was added and the cells further incubated at the same test temperatures for 16 h. Next, the cells were washed twice with Live Cell Imaging Solution to remove non-phagocyted bioparticles. The remaining phagocyted bioparticles were visualized and photographed under the Texas Red 586/647 channel using a Cytation 1 microscope fitted with a size 20 Olympus objective, No. 1220517, during which cells in the same field of view were also captured in brightfield. As a negative control we also captured untreated cells without the Zymosan Bioparticle under the same parameters.Triplicate visualizations of phagocyted zymosan particles and phagocytic cells were then used for assessment of phagocyted particles. Using the Cytation 1 cell analysis tool in the Texas red channel and brightfield we measured and calculated the total area of phagocyted zymosan particles per one cell.

However, low-temperature-phagocytosis cannot be determined using green zymosan as the fluorescence endpoint is influenced by the cultivation temperature. The principle of pHrodo zymosan measurable fluorescence signal is dependent on an intracellular endosome pH change after phagocytosis of the zymosan bioparticle (44), whereby pH can be influenced by temperature (45).

### Data analysis

Normal distribution of variables was tested using the Kolmogorov-Smirnov and Shapiro-Wilk tests. When necessary, variables were adjusted for statistical analysis using natural logarithmic transformation (ln x+1). As cell size, area and circularity variables were non-normally distributed, even after transformation, they were tested using non-parametric Kruskal-Wallis ANOVA. The influence of cultivation temperature and experimental day on total count of cells grown was tested using generalized linear models (GLM) with log-normal distribution, based on the sigma-restricted method. Distribution selection was based on model-scaled deviance values. The same method was also used to assess the influence of cultivation temperature and culture form of cell (adherent vs. suspension cells) on counts in pairs at particular cultivation temperatures. The expected interaction between temperature and culture form of cell was added into the model and Bonferroni adjustment applied, a level of significance of 0.017 being considered as statistically significant. Data from the first day of cultivation in Experiment 2 were excluded from the analysis as cell cultivation provided identical numbers. The final proportion of cells in suspension (day 6) was assessed using the difference test between proportions. The impacts of design used for cell cultivation (Experiment 1 vs. Experiment 2) and the two different freezing designs (Experiment 3) on total count of cells grown were also analyzed separately for each temperature. Phagocyte activity values (Experiment 4) were calculated from the original data with the control subtracted, the value then being relativized to 10,000 cells as the culture growing rates were different at each temperature. The moving average of two subsequent values was then used for smoothing data with high variability and the dataset obtained checked for normality and log-transformed. Univariate ANOVA was used to test for differences in phagocyte activity and the total area of phagocyted zymosan particles per one cell between temperatures, with Fisher's Least Significant Difference (LSD) *post-hoc* test used to identify which temperature pairs were statistically different. Finally, we calculated the parameters of respiratory burst based on the chemiluminescence data, i.e., time-to-start of response (T_start_), time-to-peak response (T_peak_), time-to-end of response (Tend), peak intensity (Peak) and total capacity (Integral, I) according to the equation from Heger et al. (16). In each case, statistical analysis was undertaken using the TIBCO Statistica^®^ software package v.14.0.0 (TIBCO Software Inc., Palo Alto, CA, USA).

## Results

### Experiment 1

**Figure 1:**
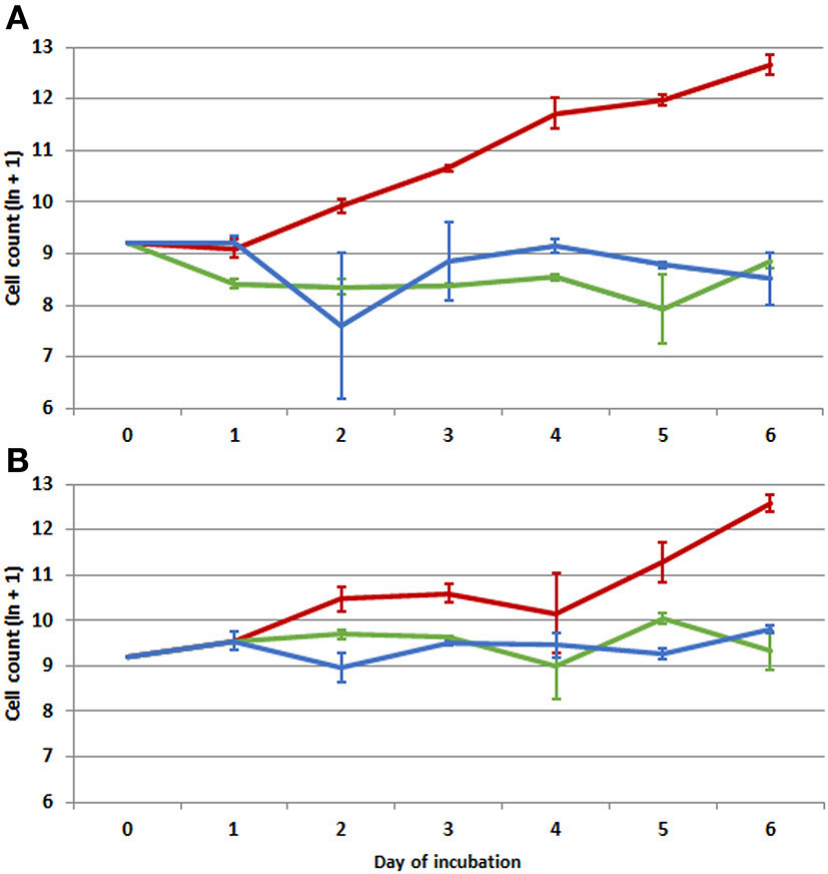
Development in counts of macrophages (ln-transformed) during **(A)** culturing at different temperatures (8, 17.5, and 37°C) in Experiment 1 and **(B)** culturing at different temperatures (8, 17.5, and 37°C) after preincubation at 37°C (0–1 day) in Experiment 2. Red line-−37°C, green line-−17.5°C, blue line-−8°C. Generalized linear models (GLM) analysis for 3 replicates/3 repetitions indicates influence of temperature (GLM, *W* = 50.74, *p* < 0.001) and cultivation day (GLM, *W* = 11.67, *p* = 0.070) on cell number in Experiment 1 and influence of temperature (GLM, *W* = 35.31, *p* < 0.001) and cultivation day (GLM, *W* = 14.00, *p* = 0.016) in Experiment 2. Average data and standard deviation (error bars) at each day of incubation shown based on 3 replicates/3 repetitions, *n* = 9.

The total count of cells grown was significantly influenced by cultivation temperature (GLM, *W* = 50.74, *p* < 0.001), with maximum growth rate at the highest temperature (Figure 1A).

**Figure 2:**
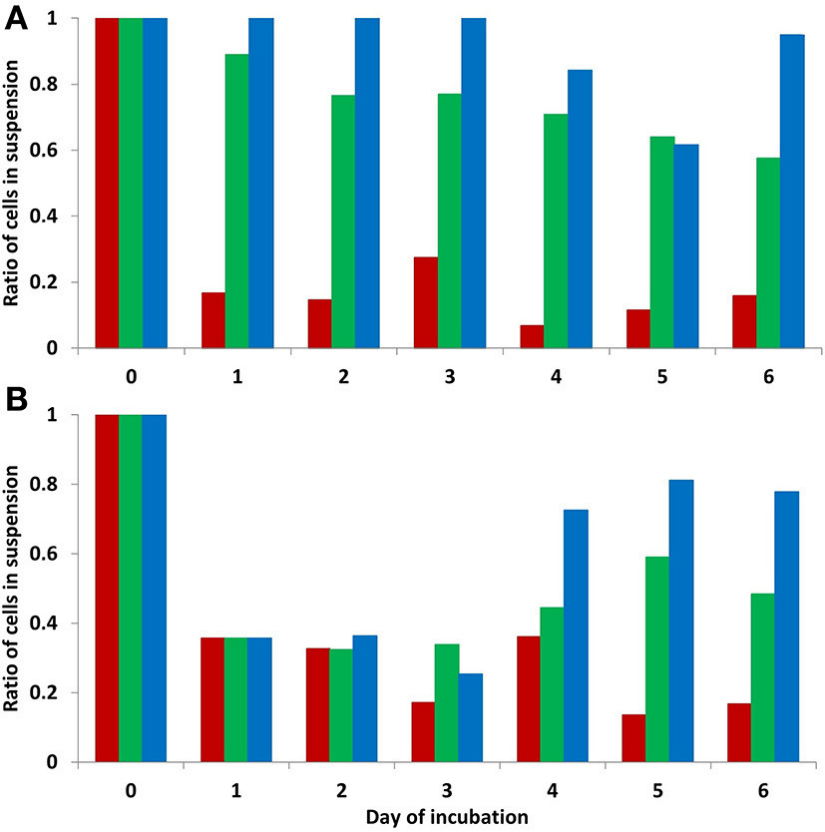
Ratio of cells in suspension vs. attached cells during 6 days of culturing at different temperatures (8, 17.5, and 37°C) in Experiment 1 **(A)** and culturing at different temperatures (8, 17.5, and 37°C) after preincubation at 37°C in Experiment 2 **(B)**. Red column-−37°C, green column-−17.5°C, blue column-−8°C. At temperature 8°C, the majority of cells were non-adherent in suspension, while attached cells prevailed at 37°C. Average data shown based on 3 replicates/3 repetitions, *n* = 9. The proportion of cells in suspension differed significantly between all three cultivation temperatures on day 6 of both experiments (difference test between proportions; *p* < 0.001).

Any influence of cultivation duration in days was not confirmed (GLM, *W* = 11.67, *p* = 0.070) as cell numbers only changed at the temperature of 37°C. Two culture type/form of cells, i.e., attached cells and non-adherent cells in suspension, showed significantly different development patterns at the lowest cultivation temperature of 8°C (Table 1). At this temperature, the majority of cells were non-adherent in suspension. While the ratio of cells in suspension compared to attached cells declined gradually at the cultivation temperature of 17.5°C and attached cells prevailed at 37°C (Figure 2A), a similarly high level of variability for cell numbers in suspension was recorded at all temperatures, albeit at different scales. The final proportion of cells in suspension (day 6) significantly differed (difference test between proportions; *p* < 0.001).

**Table 1:** Influence of cultivation temperature (8, 17.5, and 37°C) and culture type/form of cells (adherent vs. in-suspension cells) on cell proliferation pattern in Experiment 1 during 6 cultivation days.

**Cultivation temperatures**	**Effects**		
	**Temperature (temp)**	**Culture type/form of cell (form)**	Interaction temp*form
8 vs. 17.5°C	3.867 *p* = 0.049	**16.781** ***p*** **<** **0.001**	5.099 *p* = 0.024
8 vs. 37°C	**7.015** ***p*** **=** **0.008**	**9.005** ***p*** **=** **0.003**	**7.632** ***p*** **=** **0.006**
17.5 vs. 37°C	5.054 *p* = 0.025	1.514 *p* = 0.219	2.027 *p* = 0.155

Significant results are in bold.

**Figure 3:**
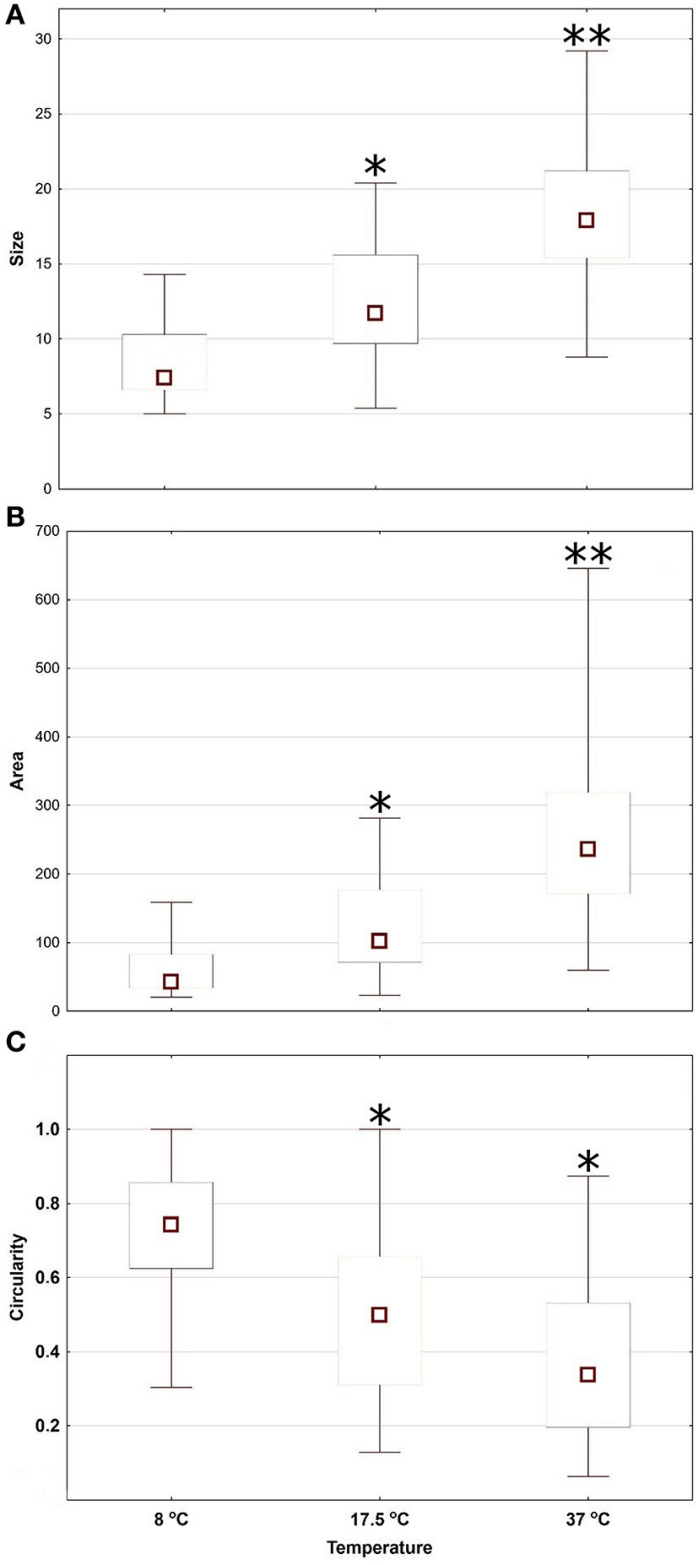
Morphological parameters (size in μm determined by fitting an ellipsis equation on the object contour—**A**, area in μm^2^ determined by counting the pixels inside the object contour—**B** and circularity index calculated using a standard ellipse formula, where 1 = a perfect circle—**C**) of macrophages cultivated at three different temperatures (8, 17.5, and 37°C) in Experiment 1. Square = median, box = inter-quartile range, whiskers = min-max range. Asterisks represent statistical significance of cell morphological parameters compared between cells cultivated at 8°C vs. cells cultivated at 17.5 and 37°C; *p < 0.05, **p < 0.01; 3 replicates/3 repetitions; univariate ANOVA with Fisher's LSD *post-hoc* test.

**Figure 4:**
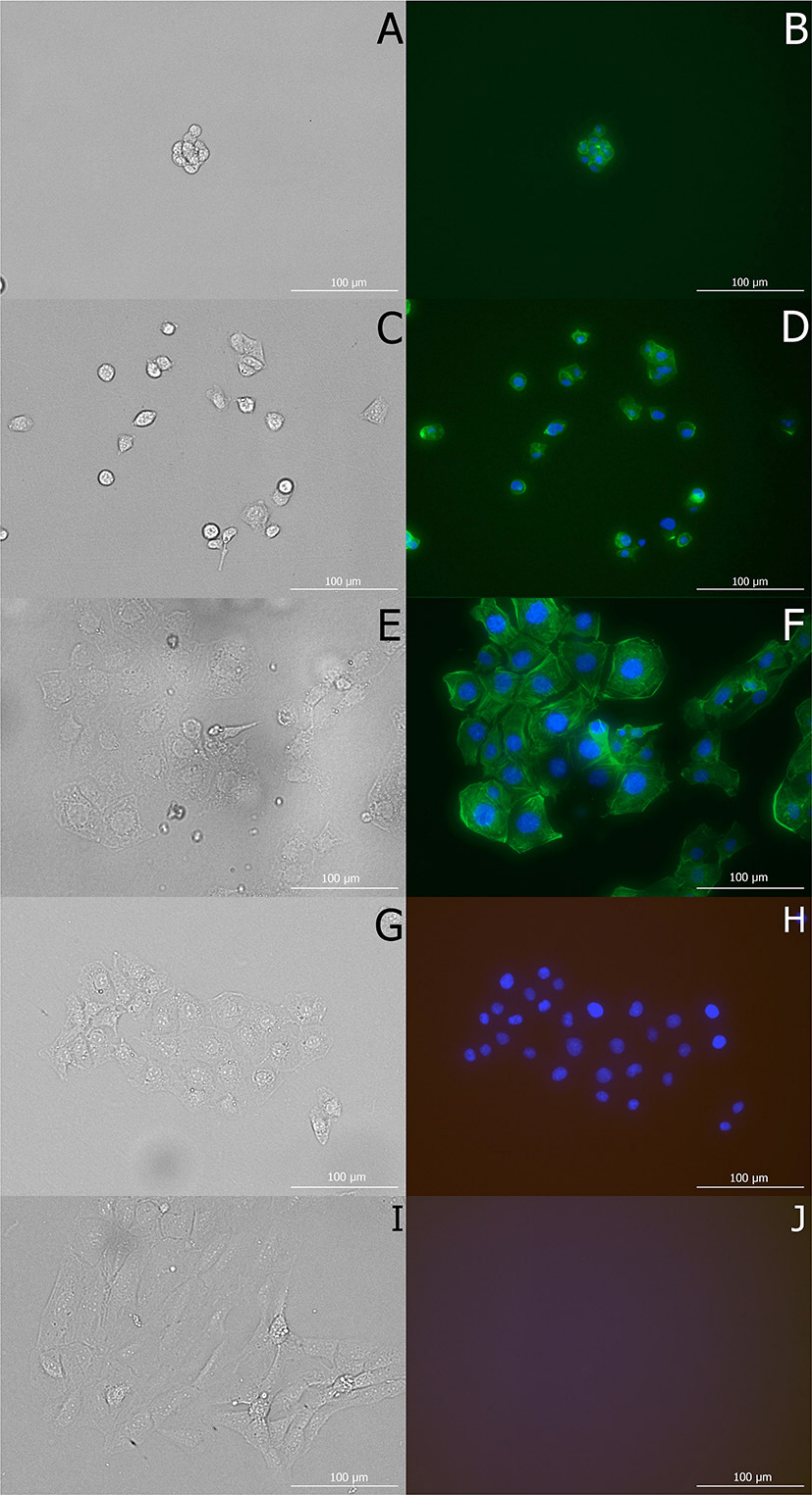
Morphology of macrophage cells *in vitro* in Experiment 1 after 48 h of incubation at test temperatures (8, 17.5, and 37°C). Cells are visualized with a Cytation 1 microscope in brightfield (**A,C,E**, respectively) and fluorescent channels (GFP, DAPI) using counterstaining of actin filaments (green) and nuclear structures/DNA (blue). Control cells, treated with DAPI for calibration, captured in bright field **(G)** and all fluorescent (Texas red, GFP, DAPI) channels **(H)** and untreated cells under the same conditions **(I,J)**.

The size and area of cells cultivated at 8°C was significantly smaller than cells cultivated at 17.5 and 37°C (Figures 3A,B). On the other hand, cells at 8°C were significantly closer to circularity (Figure 3C) and often formed small clusters. While attached cells were epithelial-like and/or spindle-shaped, cells in suspension were spheroidal (Figure 4). At 17.5°C, cells mostly showed a shrunken shape, being round and/or slightly elongated. At 37°C, cells were adhered to the cultivation surface and had usually a polygonal shape. A video sequence composed of images captured every 2 min at bright field documented that the high shape variability was induced by adherence and movement of cells and the difference in cell cycle.

### Experiment 2

When cells were allowed to attach during the first day of cultivation, the difference in total counts was significantly influenced by both temperature (GLM, *W* = 35.31, *p* < 0.001) and duration of cultivation in days (GLM, *W* = 14.00, *p* = 0.016) (Figure 1B). However, as a high percentage of cells remained attached until the third day of cultivation, the number of living cells declined less during the first day of cultivation at the lowest temperature (8°C) than in Experiment 1. Later, cells started to detach from the plate surface into suspension at both lower temperatures, resulting in ~50 and 80% of cells in suspension at 17 and 8°C, respectively (Figure 2B). The proportion of cells in suspension differed significantly between all three cultivation temperatures on day 6 of the experiment (difference test between proportions; *p* < 0.001). A different development pattern was confirmed at the higher cultivation temperature (37°C), with only 15% of detached/newly formed by cellular division cells at the end of cultivation (Table 2).

**Table 2:** Influence of cultivation temperature (8, 17.5, and 37°C) and culture type/form of cells (adherent vs. in-suspension cells) on cell proliferation in Experiment 2 during 5 cultivation days after preincubation at 37°C.

**Cultivation temperatures**	**Effects**		
	**Temperature (temp)**	**Culture type/form of cells (form)**	Interaction temp*form
8 vs. 17.5°C	1.221 *p* = 0.269	0.034 *p* = 0.854	2.100 *p* = 0.147
8 vs. 37°C	**17.040** ***p*** **<** **0.001**	1.751 *p* = 0.186	4.684 *p* = 0.030
17.5 vs. 37°C	**14.058** ***p*** **<** **0.001**	**6.380** ***p*** **=** **0.012**	1.762 *p* = 0.184

Significant results are in bold.

Counts for the two cultivation designs used in Experiment 1 and Experiment 2 differed at the lower temperatures (Table 3), with Experiment 2 producing a two to three times higher cell count by the end of cultivation. At 37°C cultivation temperature, the cell count approached 300,000 at the end of both experiments (Figure 1).

**Table 3:** Comparison of two cultivation designs (Experiment 1—cells were cultured immediately at test temperatures 8, 17.5, and 37°C vs. Experiment 2—cells were preincubated for 24 h at 37°C and then cultured at test temperatures 8, 17.5, and 37°C) on cell growth.

**Temperatures**	***W***	***p***
8°C	8.078	**0.004**
17.5°C	26.434	**<0.001**
37°C	0.104	0.748

Significant results are in bold.

W, Wald statistics.

### Experiment 3

This experiment confirmed the ability of bat-derived macrophages to survive at temperatures far below freezing point (−20°C). The percentage of survival was 28 and 37% for attached and in-suspension cells, respectively. There was no statistical difference between cell survival when freezing was initiated with cells in suspension or attached to the cultivation well surface (GLM, *W* = 1.23, *p* = 0.267); however, freezing tended to cause detachment of cells from the cultivation plate surface. Both frozen as attached and in-suspension cell cultures proved viable and proliferated 16-fold when cultivated 7 days at 37°C in the post-thaw period.

### Experiment 4

**Figure 5:**
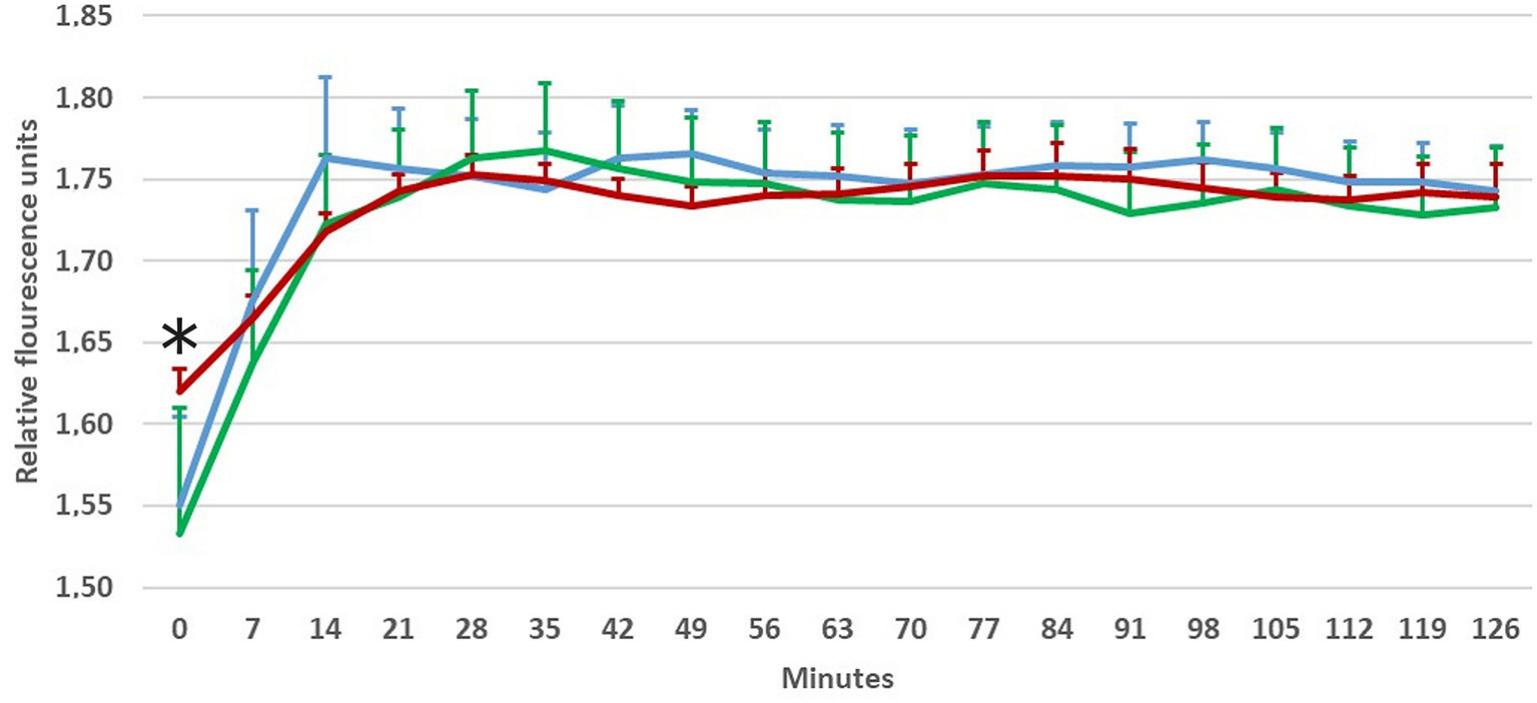
Phagocytic activity of macrophages induced by Green Zymosan Bioparticle in Experiment 4a and measured using fluorescence (Ex/Em) at 485/528 nm on a Cytation 1 every 7 min for 2 h at a temperature of 27°C. Before application of zymosan particles, cells were preincubated for 24 h at test temperatures (8, 17.5, and 37°C). Red line-−37°C, green line-−17.5°C, blue line-−8°C; *Initial levels of phagocytic activity (within 1 min after application which corresponds to time point 0) differ significantly at 8 and 17.5°C when compared with 37°C (Fisher's LSD, *F* = 5.985, *p* = 0.008). Average data and positive values of standard deviation (error bars) at each minute of measurement shown based on 2 replicates/5 repetitions, *n* = 10.

Phagocytic activity was only impacted by prior incubation temperature in the first measurement (Fisher's LSD, *F* = 5.985, *p* = 0.008), i.e., immediately after culture removal from the incubator (Figure 5). The LSD *post-hoc* test confirmed highest levels of phagocytic activity in cells cultivated at 37°C (Table 4), with initial levels of phagocytic activity at 8 and 17.5°C being 85.8 and 83.1%, respectively, of that observed at 37°C. There was no significant difference in subsequent measurements, implying that phagocytic activity returned to similar levels within 7 min of moving the cell cultures into the measurement device, which was maintained at a set temperature at 27°C. Nevertheless, phagocytic activity peaked quicker at 8°C (after 14 min) than at 17 and 37°C (both after 28 min).

**Table 4:** Comparison of phagocyte activity in the first measurement (LSD *post-hoc* test) between cells preincubated for 24 at different temperatures (8, 17.5, and 37°C) and measured at 27°C (Experiment 4).

**Temperatures**	***p***
8 vs. 17.5°C	0.519
8 vs. 37°C	**0.015**
17.5 vs. 37°C	**0.003**

Significant results are in bold.

**Figure 6:**
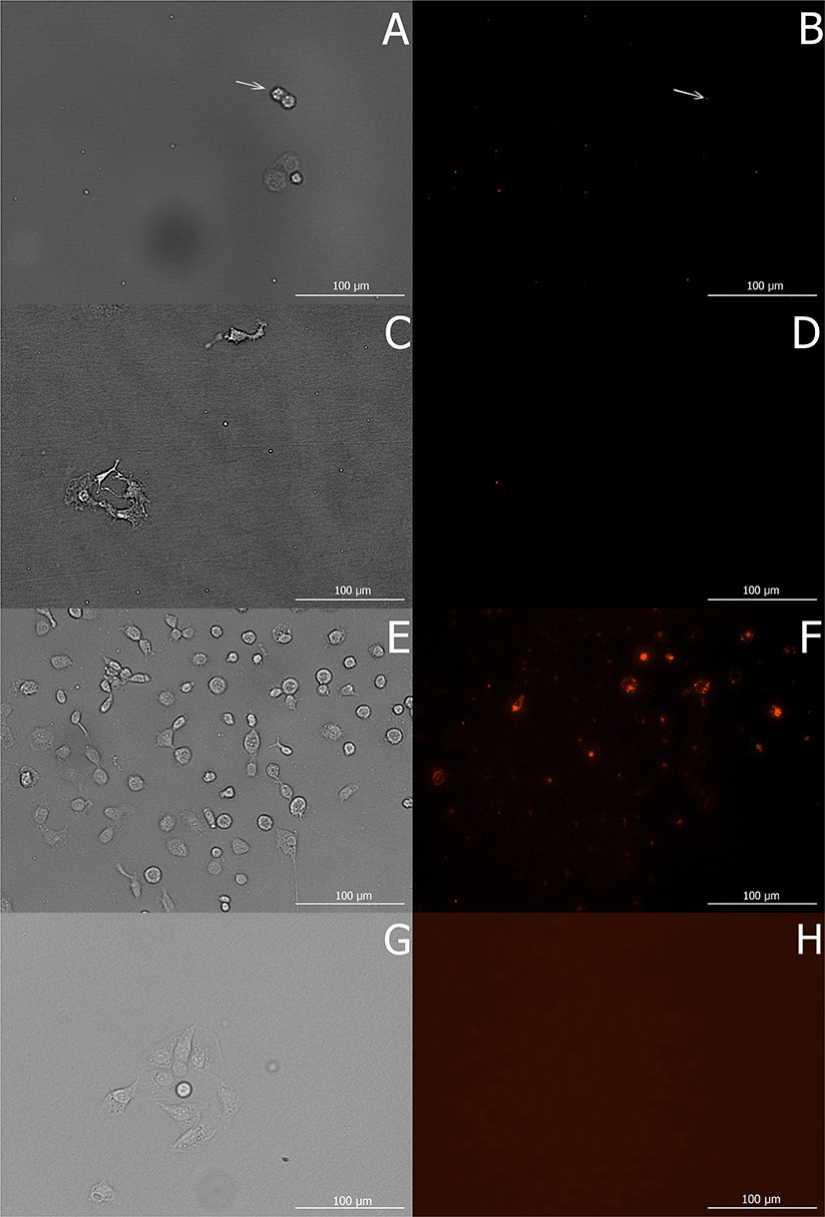
Macrophage cells *in vitro* in Experiment 4b after 16 h of phagocytic activity initiated by Red Zymosan Bioparticles at 8°C **(A)**, 17.5°C **(C)**, 37°C **(E)**, and phagocyted zymosan particles in these cells at the respective temperature conditions **(B,D,F)** visualized using Texas Red channel with a Cytation 1 microscope. At 8°C **(B)**, white arrows indicate the sporadic case of phagocyted zymosan particles within cells. Before application of zymosan particles, cells were preincubated for 48 h at test temperatures (8, 17.5, and 37°C). Untreated control cells **(G,H)** were captured under the same conditions as images **(A–F)**.

The area of phagocyted zymosan particles per one cell differed significantly between the three cultivation temperatures (Figure 6). Nevertheless, the *post-hoc* test revealed that only cells at 37°C show significantly higher phagocyte activity (Table 5).

**Table 5:** Phagocyte activity (area of phagocyted zymosan particles per one cell) of macrophages cultivated at three different temperatures (8, 17.5, and 37°C; Experiment 4).

**Temperature**	**Mean area per cell**	**SD**
8°C	1.73	1.10
17.5°C	3.13	0.56
37°C	29.95	12.42

The comparison of respiratory burst of macrophage cells in culture with values measured in whole blood samples (Table 6) showed that macrophage cultures reacted quicker (i.e., they exhibited short T_start_ and T_peak_ values) with a slightly higher level of the total activity (i.e., higher Peak and Integral values). However, they also sooner lost their phagocytic capacity (i.e., T_end_ value).

**Table 6:** Basic parameters of phagocyte respiratory burst measured in cell cultures (culture 1 and 2 correspond to average of 5 repetitions in 2 replicates) in this study and in whole blood of two bat species (16).

**Sample**	**Peak**	**Integral**	**T_start_**	**T_peak_**	**T_end_**
Culture 1	164.96	318,835.72	15.31	780.61	6,428.91
Culture 2	140.50	250,710.85	9.47	592.99	6,516.93
*Myotis myotis* (*n* = 11)	16.51	93,932.20	1,044.36	4,724.85	10,870.11
*Nyctalus noctula* (*n* = 12)	31.00	207,642.80	885.20	5,840.20	12,590.50

Peak, peak intensity of phagocyte respiratory burst; Integral, total phagocyte capacity; T_start_, time-to-start of phagocyte respiratory burst response; T_peak_, time-to-peak of phagocyte respiratory burst response; T_end_, time-to-end of phagocyte respiratory burst response (Experiment 4).

## Discussion

Macrophages include heterogeneous cell populations that may include alveolar and splenic types, Kupffer cells and microglia (29). In the present study, a peritoneal cavity-derived macrophage cell line established from a *Myotis myotis* bat was used. This cell line was established through immortalization by transfection of pRSVAg1 plasmid expressing Simian Vacuolating Virus 40 large T antigen (SV40T) as described in He et al. (43). Unlike the different phagocytes in whole blood (17), this immune-competent cell line is homogeneous, meaning that uniform and reproducible responses can be expected from the cell population under our experimental settings. Limitations of *in vitro* models, however, include a lack of complex organ systems and an inability to account for regulatory and signaling cell-to-cell interactions and biochemical/metabolic processes characteristic for different physiological states and the transitions between these states in heterotherms at the organismal level. Culturing techniques (passaging, immortalization, and long-term storage) can also affect some properties of cells (46). As a result, macrophage cell line responses may be different compared with blood-phagocytic cells *ex vivo* (16, 17). However, the total capacity of phagocytosis (Integral) *in vitro* and e*x vivo* is comparable, when we consider that the culture consists of a pure mass of professional phagocytes as opposed to different types of whole blood leukocytes.

Low temperatures are known to induces morphological changes in cells (47, 48). Here, many of the cells maintained a round or shrunken shape at low temperatures due to a reduction in their surface area (48, 49), which probably also decreases their energy costs. In our study, all cells cultivated at the lowest temperature (8°C), i.e., both those in suspension and those still adhering to the cultivation surface, had a round shape, while those held at 17.5°C mostly had a shrunken, slightly elongated, shape, rather than being round. Further, cells under optimal growth conditions adhere to the cultivation surface (50), as seen in our macrophage cell line at 37°C, while a high percentage of cells will not adhere to the cultivation surface at low temperatures. Though all cells in our Experiment 2 were pre-incubated as cells adhering to the cultivation surface, low temperatures caused them to gradually detach. However, at lower temperatures, cells maintain cell-cell adhesion, where suspended cells often formed small clusters. While adhesive cell-extracellular matrix bonds represent a dynamic reversible force, the ability to rapidly regulate adhesion and deadhesion is essential for phagocytes in defense against pathogenic agents and tissue repairing (51). Moreover, throughout the process of adhesion, the actin filament structure of the cell is often dramatically rearranged (52); therefore, healthy cells correspond to non-adherent cells = round-shape cells, but not necessarily *vice versa*. Also, the phagocytic capacity of macrophages correlates with their surface features (47), with macrophages having ruffled cell membranes performing better than those with smooth cells. Extreme membrane deformations with reorganization of cytoskeletal polymers (53) to actin-reinforced filopodia (54), lamellipodia (53) or other protrusion structures, support pathogen capture and removal (44, 53). The ability to rapidly change shape in response to activation is thus critical for phagocytosis (55), meaning that cells at 37°C have an advantage compared with cells at low temperatures, in which chilling induces actin to assemble (56) and form relatively stable round-shaped cells.

The number of white blood cells, including monocytes as macrophage precursors, decreases during bat hibernation and the number of macrophages is lower compared to active metabolism states (7, 57). In the present study, we were able to confirm that macrophages in hibernators can survive low body temperatures both *in vivo* (21) and *in vitro*. Indeed, Mazur et al. (58) demonstrated that the cells of heterothermic hibernating mammals may survive *in vitro* temperatures lower than 0°C. While this temperature drop will reduce the relative number of viable cells (48, 59); levels of protein synthesis (60), cell proliferation and differentiation (61), and cell responses to subsequent stress (62), the fact that they survive is unique. In comparison, the cells and tissues of non-hibernating mammalian species may be destroyed by short periods of exposure to temperatures below freezing point if cryoprotectants are not supplied (63, 64). In our Experiment 3, the bat-derived macrophage cell line survived freezing temperatures of −20°C with no external addition of cryoprotectant, which usually serve to maintain the viability of nucleated mammalian cells (65).

The macrophage cell line results presented here are species-specific to the greater mouse-eared bat. Unfortunately, similar immune cell lines derived from other bat species are not yet available, meaning that comparative studies are not possible. Interestingly, however, of all the European bat species, the greater mouse-eared bat displays a ca. 100% *Pseudogymnoascus destructans* fungal agent infection prevalence and the highest infection intensity (66). Thus, the results of the present *in vitro* study can be discussed in terms of what we know of white-nose syndrome infectious processes in the species the macrophage cell line was derived from. Contrary to North American bat species (66, 67), extensive damage of flight membranes is not seen in the European greater mouse-eared bat (36), most probably due to mechanisms of tolerance evolving in Palearctic bats (66). What we see on pathological examination of greater mouse-eared bats, however, are inflammatory responses in fungal infection-induced skin lesions sampled during the late-hibernation period (36). This supports our observations regarding the phagocytic cells ability to perform *in vitro*, even at lower temperatures like those encountered during torpor bouts and/or periodic arousals from hibernation. Most likely, macrophages suppressing improper immune responses (68), such as delayed-type hypersensitivity (20), and the prevailing anti-inflammatory setting of macrophages in greater mouse-eared bats (28) contribute to tolerance against white-nose syndrome in this species (66). Macrophages may also take part in the healing and remodeling of wing membrane tissues damaged by white-nose syndrome infection (24, 27, 36), supporting restoration of flight membrane integrity and functioning in the post-hibernation period.

To conclude, we show that greater mouse-eared bat-derived macrophages proliferate at low temperatures typical for heterothermic organisms in torpor, differ in their shape and ability to attach to a cultivation plate surface in a temperature-dependent manner, and are able to restore phagocytic activity within minutes of translocation into a higher temperature environment. Our *in vitro* experimental data on the macrophage cell line suggests that, while the overall performance of macrophages is suppressed at lower cultivation temperatures, these immune cells are still capable of functioning, even under harsh conditions. As such, the controlled conditions used in cell cultures appear to provide a suitable surrogate model for studying morphological and functional cellular phenomena and simulation of body-temperature-dependent changes in heterotherms.

## Data availability statement

The original contributions presented in the study are included in the article/supplementary material, further inquiries can be directed to the corresponding author.

## Author contributions

Conceptualization, methodology, and visualization: MN, JZ, VS, and JP. Formal analysis: JZ. Investigation: MN, HD, and KZ. Writing—original draft preparation: MN, VS, JZ, and JP with contributions from all authors. Funding acquisition: MN, VS, and JP. All authors have read and agreed to the published version of the manuscript.

## Funding

This research was funded by the Internal Grant Agency of the University of Veterinary Sciences Brno (Czech Republic) IGA 221/FVHE/2019. The funders had no role in the study design, data analysis, decision to publish, or preparation of the manuscript.

## Conflict of interest

The authors declare that the research was conducted in the absence of any commercial or financial relationships that could be construed as a potential conflict of interest.

## Publisher's note

All claims expressed in this article are solely those of the authors and do not necessarily represent those of their affiliated organizations, or those of the publisher, the editors and the reviewers. Any product that may be evaluated in this article, or claim that may be made by its manufacturer, is not guaranteed or endorsed by the publisher.
